# Comparison of percutaneous 60-day peripheral nerve stimulation of the lumbar medial branches to usual care with standard interventional management for chronic low back pain—a multicenter pragmatic randomized controlled trial (RESET)

**DOI:** 10.1093/pm/pnaf147

**Published:** 2025-10-25

**Authors:** Zachary L McCormick, Denise D Lester, Michael J DePalma, Christopher A Gilmore, Sean Li, Jessica B Jameson, Mehul J Desai, Tristan E Weaver, Shivanand P Lad, Scott J Davidoff, Drew M Trainor, Kasra Amirdelfan, Mitchell P Engle, Timothy R Deer, Thomas S Lee, Francesco Vetri, Meenakshi Bindal, Melissa A Tornero-Bold, Morad N Nasseri, Steven P Cohen, William H Clark, Meredith J McGee, Joseph W Boggs

**Affiliations:** Department of Physical Medicine and Rehabilitation, University of Utah School of Medicine, Salt Lake City, UT, United States; Department of Physical Medicine and Rehabilitation, Central Virginia VA Health System, Richmond, VA, United States; Virginia iSpine Physicians, Richmond, VA, United States; Center for Clinical Research, Winston Salem, NC, United States; Premier Pain Centers, Shrewsbury, NJ, United States; Axis Spine Center, Coeur d’Alene, ID, United States; International Spine, Pain & Performance Center, Washington, DC, United States; Department of Anesthesiology, The Ohio State University, Columbus, OH, United States; Department of Neurosurgery, Duke University, Durham, NC, United States; Main Line Spine, King of Prussia, PA, United States; Denver Spine & Pain Institute, Greenwood Village, CO, United States; Department of Clinical Research, Boomerang Healthcare, Walnut Creek, CA, United States; Institute of Precision Pain Medicine, Corpus Christi, TX, United States; The Spine and Nerve Center of the Virginias, Charleston, WV, United States; Rehabilitation Institute at Sinai, LifeBridge Health, Baltimore, MD, United States; Millennium Pain Center, Bloomington, IL, United States; Department of Physical Medicine and Rehabilitation, Central Virginia VA Health System, Richmond, VA, United States; Department of Anesthesiology, The Ohio State University, Columbus, OH, United States; Department of Clinical Research, Boomerang Healthcare, Walnut Creek, CA, United States; Department of Anesthesiology, Northwestern University, Chicago, IL, United States; Department of Anesthesiology, Walter Reed National Military Medical Center, Bethesda, MD, United States; Research and Development, SPR, Cleveland, OH, United States; Research and Development, SPR, Cleveland, OH, United States; Research and Development, SPR, Cleveland, OH, United States

**Keywords:** pain management, back pain, chronic pain, low back pain, interventional, controlled trial, medial branch

## Abstract

**Objective:**

Management of refractory chronic low back pain (CLBP) includes a range of treatments (eg, physical therapy, injections, ablations, neurostimulation, surgery) with varying utilization and effectiveness. This pragmatic randomized controlled trial (RCT) evaluated the effectiveness of one treatment option, percutaneous 60-day peripheral nerve stimulation (PNS), compared to usual care with standard interventional management for CLBP.

**Methods:**

Two hundred thirty patients with CLBP were randomized in a 1:1 ratio to Group 1 (percutaneous 60-day PNS) or Group 2 (physician-directed usual care with standard interventional management). The Primary Clinical Endpoint evaluated the proportion of participants with ≥50% reductions in CLBP at 3 months post-treatment compared to baseline.

**Results:**

At the Primary Endpoint, a greater proportion of participants receiving percutaneous 60-day PNS (55%; *n* = 112; 95% confidence interval [CI] = 45-65) experienced ≥50% pain relief compared to usual care with standard interventional management (26%; *n* = 110; 95% CI = 17-34; *P* < .001). Concordant with the Primary Endpoint, percutaneous 60-day PNS also produced greater improvements in patient-centric secondary endpoints, including disability, pain interference, health-related quality of life, and analgesic consumption. Reductions in pain and resulting improvements in function were sustained through 6 months with percutaneous 60-day PNS.

**Conclusions:**

This prospective multicenter pragmatic RCT met its Primary Clinical Endpoint and found that more participants with CLBP reported pain relief at 3 months after receiving percutaneous 60-day PNS as compared to usual care with standard interventional management. Participants treated with 60-day PNS showed greater reductions in pain and more substantial improvements in functional outcomes through 6 months.

**ClinicalTrials.gov:**

NCT04246281; Primary funding: Department of Defense (DoD), with additional funding needed to complete the study provided by the study’s sponsor, SPR.

The protocol is available on ClinicalTrials.gov. The statistical analysis plan and results will be made available within 12 months of the study’s completion.

## Teaser

This pragmatic RCT evaluated the effectiveness of percutaneous 60-day peripheral nerve stimulation (PNS) compared to usual care with standard interventional management for chronic low back pain. More participants receiving percutaneous 60-day PNS (55%; n=112; 95%CI= 45, 65) experienced ≥50% pain relief compared to usual care with standard interventional management (26%; n=110; 95%CI = 17, 34; p<0.001). Reductions in pain and resulting improvements in function were sustained through 6 months with percutaneous 60-day PNS.

## Introduction

Chronic low back pain (CLBP) causes functional limitations (eg, pain-related disability and interference with activities of daily living) and reduces quality of life.^1^^,^^2^ CLBP can be difficult to treat, with a wide range of mechanisms that encompass nociceptive, neuropathic, and nociplastic processes. Usual care for CLBP (eg, physical therapy, injections, ablations, neuraxial stimulation, and surgery) is typically directed by diagnostic imaging, patient presentation, and disease burden. Shared decision making between physicians and patients is often used to determine the most appropriate plan for each clinical scenario with comparative evidence from randomized controlled trials (RCTs) typically serving as the foundation for best-practice care.

Percutaneous 60-day peripheral nerve stimulation (PNS) has been shown to produce clinically significant pain relief for various pain conditions in prospective clinical trials (see review^3^) with results corroborated by real-world evidence.^4^ Among prior studies, placebo-controlled trials comparing percutaneous 60-day PNS to sham stimulation have been successful in treating neuropathic pain in amputees (*n* = 28 participants^5^), post-surgical pain in the shoulder, knee, and foot/ankle (*n* = 66 participants^6^), and intractable knee pain (*n* = 41 participants^7^). Among patients with CLBP, 60-day PNS of the lumbar medial branch nerves has the potential to provide relief in a heterogenous patient population.^8^ A prior prospective single-arm trial (*n* = 74 participants) supported the use of 60-day PNS for CLBP, with 77% of participants experiencing meaningful improvement in pain and/or function at 14 months.^9^ Among responders who later completed a long-term follow-up survey, 65% (*n* = 23) continued to report relief at an average of 4.7 years after 60-day PNS.^10^ These findings supported the need for additional evidence, including larger sample sizes with long-term follow-up. The study design for this RCT was developed in direct response to a grant solicitation by the Department of Defense (DoD), whose stated goals included conducting comparative studies evaluating the effectiveness of different pain management strategies, with focus on research on lower back pain strategies to prevent surgery and recurrence of symptoms. To inform clinical decision making, this study design evaluated comparative effectiveness with usual care including standard interventions in an insured population seeking care for recalcitrant CLBP, aiming to generate evidence relevant to real-world treatment decisions. Therefore, the primary objective of this comparative-effectiveness RCT was to compare the proportion of participants achieving a ≥50% reduction in CLBP between those treated with percutaneous 60-day PNS (Group 1) and those receiving usual care with standard interventional management (Group 2), using sequential non-inferiority testing, which if successfully confirmed, would be followed by superiority testing.

## Methods

This study received initial institutional review board (IRB) approval from a central IRB (Western IRB, now WCG IRB, Princeton, NJ; ID: 20192897) on December 30, 2019, and was registered on ClinicalTrials.gov/study/NCT04246281 on January 29, 2020. The Study Protocol is available on the ClinicalTrials.gov registration page. Twenty-one academic and private practice sites from all regions of the United States were selected for participation. The first participant was enrolled on July 20, 2020. All methods and procedures followed the principles of the Declaration of Helsinki. Patients with CLBP confined to the lumbar region without radiation to the extremities were screened for eligibility and participants provided written, informed consent prior to study procedures. The main text of this manuscript summarizes key methodological elements relevant to the analyses, while Appendices S1-S3 contain additional information regarding Recruitment and Assessment, Primary Safety Endpoint, Statistical Methods, Study Population and Baseline Demographics, Secondary Endpoints, Observed Data, Subgroup Analysis, and Adverse Events.

### Eligibility criteria

Participants were required to have moderate to severe refractory CLBP lasting longer than 6 months, predominantly confined to the lumbar region. Key inclusion criteria included previous use of at least 2 types of pain therapies (eg, narcotic and/or non-narcotic medication, massage therapy, physical therapy, injections, ablations), active health insurance, and a stable pain treatment regimen for at least 4 weeks. Key exclusion criteria included radicular leg pain, body mass index >40, radiofrequency ablation of the lumbar medial branches within the last 6 months, prior lumbar spine surgery, or medical conditions that are contraindicated for the PNS device. A full list of eligibility criteria is shown in Appendix S1 (additional details on recruitment and eligibility assessment are also included in Appendix S2). Following the screening visit, eligible participants completed a 7-day written diary of daily average back pain intensity (Brief Pain Inventory Short Form, Question 5, BPI5; ie, average pain intensity over past 24 hours). Final eligibility required a mean BPI5 (ie, average of the 7 daily scores) of ≥4.

### Randomization and SOT

Participants were randomized using a 1:1 block randomization scheme (electronically generated permuted blocks of 2 or 4 with site stratification) to either Group 1 (percutaneous 60-day PNS) or Group 2 (physician-directed usual care with standard interventional management). Group 1 received a prognostic medial branch block (MBB) followed by percutaneous PNS lead implantation at least 1-week post-MBB, regardless of MBB outcome. Group 1’s MBB was included as a secondary outcome to reflect routine diagnostic practices and assess its prognostic value for identifying percutaneous 60-day PNS responders; results will be reported in a future manuscript. Group 2 received physician-directed usual care which included diagnostic testing (eg, MBB, imaging, other diagnostic testing) and/or interventions (eg, epidural steroid injections, trigger point injections, facet injections, sacroiliac joint injections, radiofrequency ablation, neuraxial stimulation, surgical procedures, and other usual care, such as pharmacotherapy, integrative treatments or physical therapy) for their CLBP, without limitation. For all participants, the “start of treatment” (SOT) was defined as the initial date of intervention (eg, date of percutaneous PNS lead implantation in Group 1, or date of the first intervention, such as radiofrequency ablation, injection, other treatment, or start of usual care, for Group 2). All subsequent follow-up visits are reported in reference to the SOT date (eg, 3 months post-treatment refers to 3 months after SOT).

### Data management and blinding

Due to the nature of the randomized treatment assignments (ie, percutaneous 60-day PNS or pragmatic treatment via usual care with standard interventions and the heterogeneity of treatments that could be utilized by a participant), blinding was not possible following randomization assignment. All study assessments and data collection were conducted by trained study personnel (eg, physician, nurse, or study coordinator) at independent academic or private practice research centers. All statistical analyses were conducted by an independent third-party. Medication reviews were conducted by a blinded panel of 3 physicians who were not involved in the study. An Independent Medical Reviewer adjudicated adverse events.

### Healthcare resource utilization

Each participating site documented all low back pain healthcare resource utilization by each participant (ie, use of any diagnostic testing and treatments that could affect CLBP) throughout study participation. Consistent with the pragmatic design of the study, all participants (ie, those in both groups) were eligible to receive additional treatments for CLBP as needed at any point throughout study participation, as determined by a study investigator or the participant’s physician. All participants initially randomized to Group 2, usual care with standard interventional management, were offered the opportunity to cross over to receive percutaneous 60-day PNS at 12 months after SOT (ie, regardless of improvement or treatments received during the first 12 months of participation).

### Percutaneous 60-day PNS lead implantation

**Figure 1.**
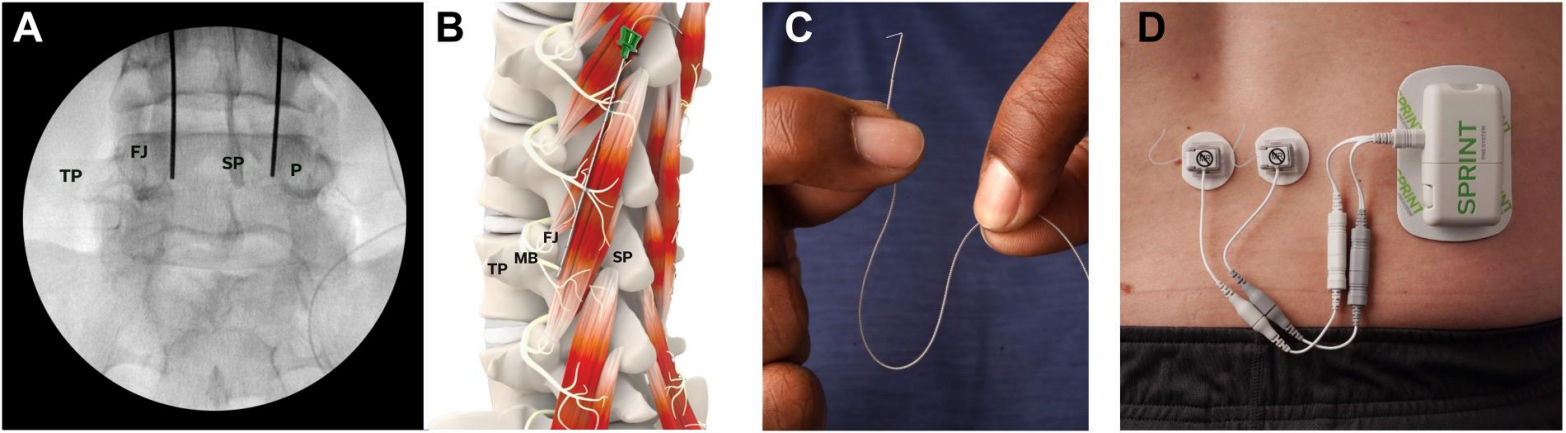
Percutaneous 60-day peripheral nerve stimulation (PNS) targeting the medial branch nerves via fine-wire coiled leads for the treatment of chronic low back pain (CLBP). Participants underwent percutaneous 60-day PNS lead implantation targeting the medial branches of the lumbar dorsal rami at the spinal level in the center of the region of axial back pain under ultrasound and/or fluoroscopic guidance. (A) An anteroposterior (AP) fluoroscopic view shows the stimulation test probe insertion targeting the medial branches of the dorsal rami at a location over lamina, medial and inferior to the facet joint (prior to deployment of the percutaneous lead). (B) An oblique view of paraspinal anatomy demonstrates the approach for Percutaneous PNS lead implantation targeting the lumbar medial branch nerves over lamina medial and inferior to the facet joint, prior to innervation of the multifidus muscles. (C) The fine-wire, coiled percutaneous leads (MicroLead open-coil PNS leads, SPR Therapeutics, Inc. [“SPR”]) have a slim profile (300 µm outer diameter) and a large (1.5 cm long) monopolar stimulating electrode. The flexible lead was designed specifically for percutaneous use to conform to patient movement. (D) The percutaneous 60-day PNS system (SPRINT PNS System, SPR) included a body-worn stimulator that was controlled via wireless Bluetooth remote and coupled to 2 percutaneous leads implanted in the low back, targeting the lumbar medial branch nerves. Abbreviations: FJ, facet joint; MB, medial branch; P, pedicle; SP, spinous process; TP, transverse process. Images used with permission from SPR.

Group 1 participants received an FDA 510(k) cleared (approved) device, the SPRINT PNS System (SPR Therapeutics, Inc. [“SPR”], Cleveland, OH), used on-label for the treatment of chronic pain in the low back. Percutaneous MicroLead open-coil PNS leads (SPR, Figure 1) were implanted under ultrasound and/or fluoroscopic guidance targeting the medial branches of the dorsal rami^8^ over lamina, medial and inferior to the facet joint at the lumbar level in the center of the region of pain. Most leads were placed 1-2 cm lateral from midline at a cephalocaudal angle of approximately 30-60° relative to skin at a depth of 5-8 cm depending on body habitus, with the electrode tip designed to be placed remotely from the nerve (ie, ideally 0.5-1.0 cm).^8^^,^^11^ Successful lead implantation targeting the medial branch nerves was confirmed by visualization of multifidus muscle tension with ultrasound imaging and by participant-reported comfortable sensations overlapping their area of pain. Percutaneous leads were secured with surgical glue and covered by waterproof dressings. A small external pulse generator was connected to the percutaneous leads and delivered stimulation to generate comfortable, cyclic activation of the multifidi (stimulation parameters: frequency: 12 Hz; duty cycle: 50%; amplitude range: 0-30 mA; pulse duration range: 10-200 µs). All participants were instructed to use PNS at an intensity that generated comfortable sensations in their low back for 6-12 hours per day, while continuing most of their normal activities. Leads were removed using gentle traction in an outpatient procedure after up to 60 days.

### Outcome measures

Data collection and monitoring are complete through 6 months post-treatment (6 months after start of treatment for each group), including the prospectively defined Primary Clinical Endpoint at 3 months post-treatment. Follow-up visits through 3 years remain ongoing. One week before each visit, participants recorded average pain intensity using a 7-day diary of daily recordings (Brief Pain Inventory Short Form, Question 5; BPI5 calculated as the mean of 7 daily scores). The study’s Primary Clinical Endpoint was prospectively defined as the proportion of participants who experienced clinically substantial reductions in average CLBP, calculated as a ≥50% reduction in average pain intensity (BPI5) at 3 months post-treatment compared to baseline. The Primary Safety Endpoint was prospectively defined as the occurrence and type of study-related adverse events (AEs; additional details on safety data collection are found in Appendix S2).

Secondary Clinical Endpoints were assessed using validated instruments completed at baseline and during follow-up visits. The mean point change, percent change, and proportion of responders (ie, ≥30% and ≥50% improvement) of each group were evaluated for average pain intensity (BPI5) and Worst Back Pain Intensity (BPI3), as well as the functional outcomes of back pain-related disability (Oswestry Disability Index; ODI) and pain interference with daily activities (Brief Pain Inventory Short Form, Question 9; BPI9). To provide additional opportunities for participants to describe improvement beyond pain relief alone, composite outcomes requiring ≥30% or ≥50% improvements in 1 of 2 domains (“and/or”) were assessed for average pain (BPI5) and/or disability (ODI) as well as average pain and/or pain interference (BPI9). To provide more rigorous assessment requiring improvement in multiple domains, composite outcomes requiring ≥30% improvements in both domains (“and”) were assessed for average pain and disability as well as average pain and pain interference. Patient global impression of change (PGIC) measured on a 7-point (-3, “Very Much Worse” to +3, “Very Much Improved”) Likert scale was assessed by evaluating the mean change in improvement, as well the proportion with +1, +2, and +3 improvement (eg, proportion with ≥1 Minimally Improved PGIC). Finally, health-related quality of life (HRQoL), as determined by the EuroQol Visual Analog Scale (EQ-VAS) was assessed by evaluating the mean point change and mean percent change. Participants recorded any analgesic medication usage in weekly diaries at baseline and follow-up. A review committee of 3 blinded physicians assessed analgesic consumption by independently scoring changes as a clinically meaningful “Increase,” “Decrease,” or “No Change” from baseline based on their clinical judgement and assessment of all medications and dosages consumed in the medication diary, with final categorization based on majority rule.

### Statistical methods

Description of the power analysis and handling of missing diary entries are provided in Appendix S2. Participants who were randomized and continued to meet all eligibility criteria prior to treatment were included in the Full Analysis Set following intention-to-treat principles, which is the analysis set described in this report (unless otherwise noted). To account for dropouts, the Primary Clinical Endpoint and applicable secondary endpoints were analyzed with imputation using predictive mean matching, where a simulated regression model derived missing data.^12^^,^^13^ The variables included in the model were average scores at each visit, as well as treatment(s) utilized (eg, 60-day PNS, RFA, Injection, etc). The results from each imputed endpoint were pooled across 50 datasets to ensure robustness.

The Primary Clinical Endpoint was tested using the Newcombe Hybrid Score Method under a closed procedure of sequential non-inferiority and superiority testing using alpha levels of 0.025 (1-sided) and 0.05 (2-sided), respectively. Under the sequential testing, non-inferiority was first assessed by calculating the 1-sided 97.5% confidence interval (CI) for the difference in proportions (usual care with standard interventional management minus 60-day PNS). Non-inferiority of 60-day PNS was confirmed if the 1-sided CI was less than 10%, the pre-specified non-inferiority margin. If non-inferiority was established, superiority of 60-day PNS was confirmed if the entire 2-sided 95% CI for the difference was below zero, the pre-specified superiority margin. *P* values were derived by testing the difference in proportions (pooled across 50 datasets) between the 2 treatment groups. Two-sample Wilcoxon rank-sum tests were used to determine changes from baseline (ie, point change and percent change) and the Exact Mantel Haenszel Chi-Square test was used to determine significant differences in categorical data. For raw continuous data, a mixed model ANOVA assessed group-level (percutaneous 60-day PNS vs usual care with standard interventional management) differences for each outcome measure using completed timepoints (baseline, 1-, 2-, 3-, and 6-month post start of treatment). When significant differences were identified, post-hoc comparisons were conducted using least square means and their associated 95% confidence intervals to assess group differences at each completed time point, while accounting for baseline scores. An alpha level <0.05 was considered to be statistically significant unless otherwise noted. Data are reported as mean ± standard deviation unless otherwise noted. All statistical analyses for primary and secondary endpoints were performed by an independent biostatistics group (WCG Statistics Collaborative, Washington, DC) using SAS software (SAS Institute Inc., Cary, NC, USA).

Results from the observed dataset (ie, participants who completed the visits, without imputation for missing data) for the Primary Clinical Endpoint are reported in Appendix S2, along with worst-case (impute all missing data as failures) and best-case (impute all missing data as successes) scenarios, secondary outcomes, and prespecified subgroup analyses comparing Primary Clinical Endpoints for interventions commonly received as part of usual care with standard interventional management (eg, PNS vs RFA, PNS vs injections, PNS vs spinal cord stimulation, etc).

## Results

### Study population

**Figure 2.**
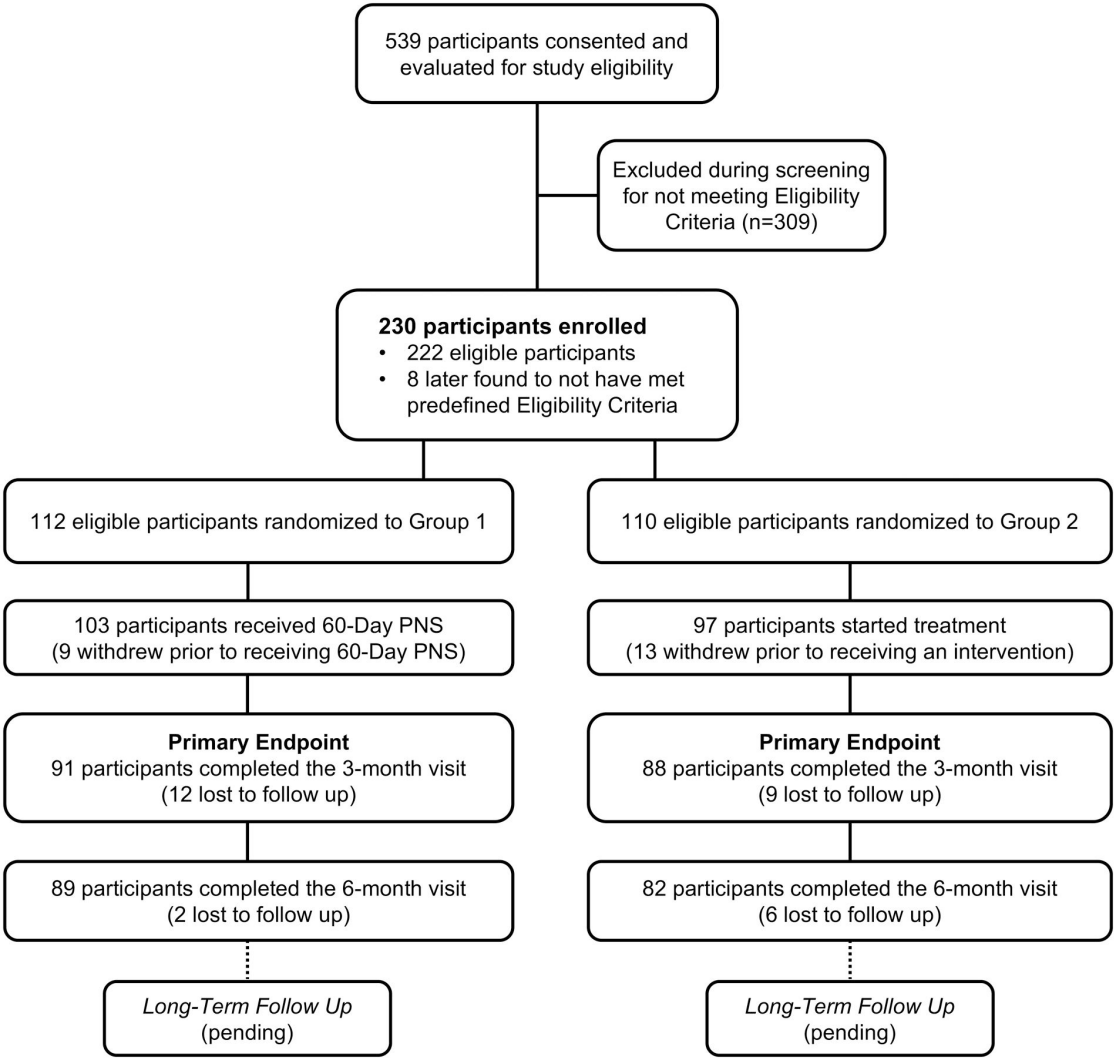
Participant flow diagram. Participants with axial CLBP were consented, evaluated for eligibility, enrolled, randomized (1:1 to Group 1—percutaneous 60-day PNS, or Group 2—usual care with standard interventional management), and started treatment for their CLBP. Of the 230 participants who were enrolled (randomized), 8 were excluded from the Full Analysis set after monitoring identified they did not meet the prospectively defined eligibility criteria during enrollment (late ineligibles, eg, started new medication within 4 weeks of baseline, steroid injection within 3 months of baseline, or pending litigation for workers compensation or other secondary gain issues, etc). Following randomization, but prior to receiving treatment, 9 participants in Group 1 and 13 participants in Group 2 withdrew their study participation. The Primary Endpoint occurred at 3 months after the start of treatment (SOT). A total of 179 participants, 91 in Group 1 and 88 in Group 2, successfully completed the visit and were included in the analysis (note, at the 3-month visit, one Group 1 participant who provided scores for some outcome measures had missing pain scores and therefore was not included in the Primary Endpoint analysis). Data collection through the 6-month visit is complete, with prospective follow-up visits ongoing for later visits. Participants in Group 2 are offered the option to crossover to receive percutaneous 60-day PNS at 12 months post-treatment. Abbreviations: PNS, peripheral nerve stimulation; Group 1, percutaneous 60-day PNS; Group 2, usual care with standard interventional management.

Between July 2020 and December 2023, 539 patients were screened for eligibility, with 230 participants randomized. Among these, 8 participants were found during data monitoring to not meet the prospectively defined Eligibility Criteria (eg, late “ineligibles” because they received an intervention within the baseline treatment window or were found have ongoing litigation for worker’s compensation) and were excluded from analysis. A total of 179 participants, 91 in Group 1 and 88 in Group 2, successfully completed the Primary Endpoint visit 3 months post-treatment, with 171 participants, 89 in Group 1 and 82 in Group 2, completing the 6-month visit. A CONSORT flow diagram is provided in Figure 2. Baseline demographics for participants in the Full Analysis Set are reported in Table 1, with additional information provided in Appendix S2. Group means and comparative statistics for each endpoint at baseline and follow-up are reported in Table 2.

**Table 1.** Demographics and baseline characteristics.

	Percutaneous 60-day PNS (Group 1, *n* = 112)	Usual care with standard interventional management (Group 2, *n* = 110)	Total (*n* = 222)
***Participant demographic and clinical characteristics***			
Age (years), mean ± SD	55 ± 14	52 ± 13	53 ± 14
BMI, mean ± SD	29 ± 5.5	30 ± 5.4	29 ± 5.5
CLBP duration (years), mean ± SD	14 ± 13	14 ± 12	14 ± 13
Female, % (*n*)	53 (59)	51 (56)	52 (115)
***Diagnosis or cause of CLBP***			
Lumbar spondylosis/Facetogenic pain, % (*n*)	59 (66)	54 (59)	56 (125)
Degenerative disc disease/Discogenic pain, % (*n*)	45 (50)	31 (34)	38 (84)
Nonspecific low back pain, % (*n*)	0.9 (1)	3.6 (4)	2.3 (5)
Other (eg, injury)/Unknown, % (*n*)	6.3 (7)	5.5 (6)	5.9 (13)
***Baseline employment and military service status***			
U.S. Military Service (Active Duty or Veteran), % (*n*)	22 (25)	26 (29)	24 (54)
Currently working, % (*n*)	58 (65)	56 (62)	57 (127)
Retired (not due to health), % (*n*)	26 (29)	23 (25)	24 (54)
Disabled and/or retired because of low back pain, % (*n*)	8.0 (9)	3.6 (4)	5.9 (13)
Unemployed, % (*n*)	3.6 (4)	9.1 (10)	6.3 (14)
Other (eg, homemaker, on leave of absence, student, other), % (*n*)	6.3 (7)	9.1 (10)	7.7 (17)
***Baseline scores and medication consumption***			
Average pain intensity, BPI5, mean ± SD	6.0 ± 1.2	6.1 ± 1.3	6.1 ± 1.2
Oswestry Disability Index (% Point), ODI, mean ± SD	42 ± 16	43 ± 16	43 ± 16
Pain interference, BPI9, mean ± SD	6.2 ± 1.4	6.4 ± 1.6	6.3 ± 1.5
Worst pain intensity, BPI3, mean ± SD	7.4 ± 1.2	7.5 ± 1.2	7.5 ± 1.2
Depression, BDI-II, mean ± SD	6.8 ± 5.8	8.0 ± 5.5	7.4 ± 5.7
Health-related quality of life, EQ-VAS, mean ± SD	66 ± 18	61 ± 18	64 ± 18
Participants reporting analgesic consumption for back pain, % (*n*)	63 (71)	67 (74)	65 (145)
Opioid consumption among those taking opioids, MME (*n*)	26 (14)	26 (13)	26 (27)

Results shown as mean ± SD unless otherwise stated.

Abbreviations: BDI-II, Beck Depression Inventory; BMI, body mass index; BPI, Brief Pain Inventory; BPI3, worst pain intensity; BPI9, pain interference; CLBP, chronic low back pain; EQ-VAS, EuroQol Visual Analog Scale; MME, milligrams morphine equivalent; ODI, Oswestry Disability Index; PNS, peripheral nerve stimulation; SD, standard deviation.

**Table 2.** Means for each endpoint.

	Percutaneous 60-day PNS (Group 1, *n* = 112)		Usual care with standard interventional management (Group 2, *n* = 110)		*P*
	LS Mean	95% CI	LS Mean	95% CI	
***Average pain intensity (BPI5)***					
Baseline	6.0	5.8-6.3	6.1	5.9-6.3	N/A
1 month	3.5	3.1-4	4.6	4.2-5	<.001
2 months	2.8	2.4-3.2	4.6	4.2-5.1	<.001
3 months	3.0	2.5-3.4	4.5	4-4.9	<.001
6 months	3.6	3.1-4	4.5	4.1-5	.004
***Oswestry Disability Index (ODI)***					
Baseline	42	39-45	43	40-46	N/A
1 month	26	23-29	33	30-36	.003
2 months	22	19-25	32	29-35	<.001
3 months	22	19-25	30	27-33	<.001
6 months	24	21-27	31	28-34	.004
***Pain interference (BPI9)***					
Baseline	6.2	5.9-6.5	6.4	6.1-6.6	N/A
1 month	3.3	2.9-3.8	4.4	3.9-4.8	.003
2 months	2.7	2.2-3.1	4.3	3.8-4.7	<.001
3 months	2.7	2.1-3.2	4.2	3.7-4.7	<.001
6 months	3.1	2.6-3.6	4.4	3.9-4.9	<.001
***Worst pain intensity (BPI3)***					
Baseline	7.4	7.2-7.6	7.5	7.3-7.7	N/A
1 month	4.8	4.4-5.3	5.9	5.5-6.4	.001
2 months	3.9	3.5-4.4	5.9	5.5-6.4	<.001
3 months	4.0	3.5-4.5	6.0	5.4-6.5	<.001
6 months	4.7	4.2-5.3	6.1	5.5-6.7	.001

Post-hoc comparisons were conducted using least square means and their associated 95% confidence intervals to assess group differences at each completed time point, while accounting for baseline scores in the model.

Abbreviations: BPI, Brief Pain Inventory; BPI3, worst pain intensity; BPI5, average pain intensity; BPI9, pain interference; LS mean, least square means; ODI, Oswestry Disability Index; PNS, peripheral nerve stimulation; 95% CI, 95% confidence interval.

### Diagnostic procedures and treatments

Of the 222 randomized participants within the full analysis set, 103 participants in Group 1 and 97 participants in Group 2 continued participating in the study and made it to the start of treatment visit. No Group 1 participants who continued study participation declined 60-day PNS. In Group 1, aside from receiving the randomized treatment (ie, percutaneous 60-day PNS), no new interventions were reported prior to the Primary Endpoint. However, *n* = 6/103 Group 1 participants reported use of non-interventional treatments (eg, physical therapy, massage therapy), 4 of which began before enrollment. Group 2 diagnostic and interventional procedure utilization is shown in Table S1, with the most common intervention being radiofrequency ablation (RFA; 41%; *n* = 40/97), followed by injections (37%; *n* = 36/97; eg, epidural steroid injections, trigger point injections, facet injections, sacroiliac joint injections) and other usual care (eg, physical therapy, 17%; *n* = 16/97; chiropractic treatment; 7%; *n* = 7/97). While investigators recommended interventional procedures for each participant, a subset of participants declined and elected to defer any new treatments (9%; *n* = 9/97) or to use only oral analgesic medications for their back pain (6%; *n* = 6/97).

### Primary clinical endpoint: average pain intensity

**Figure 3.**
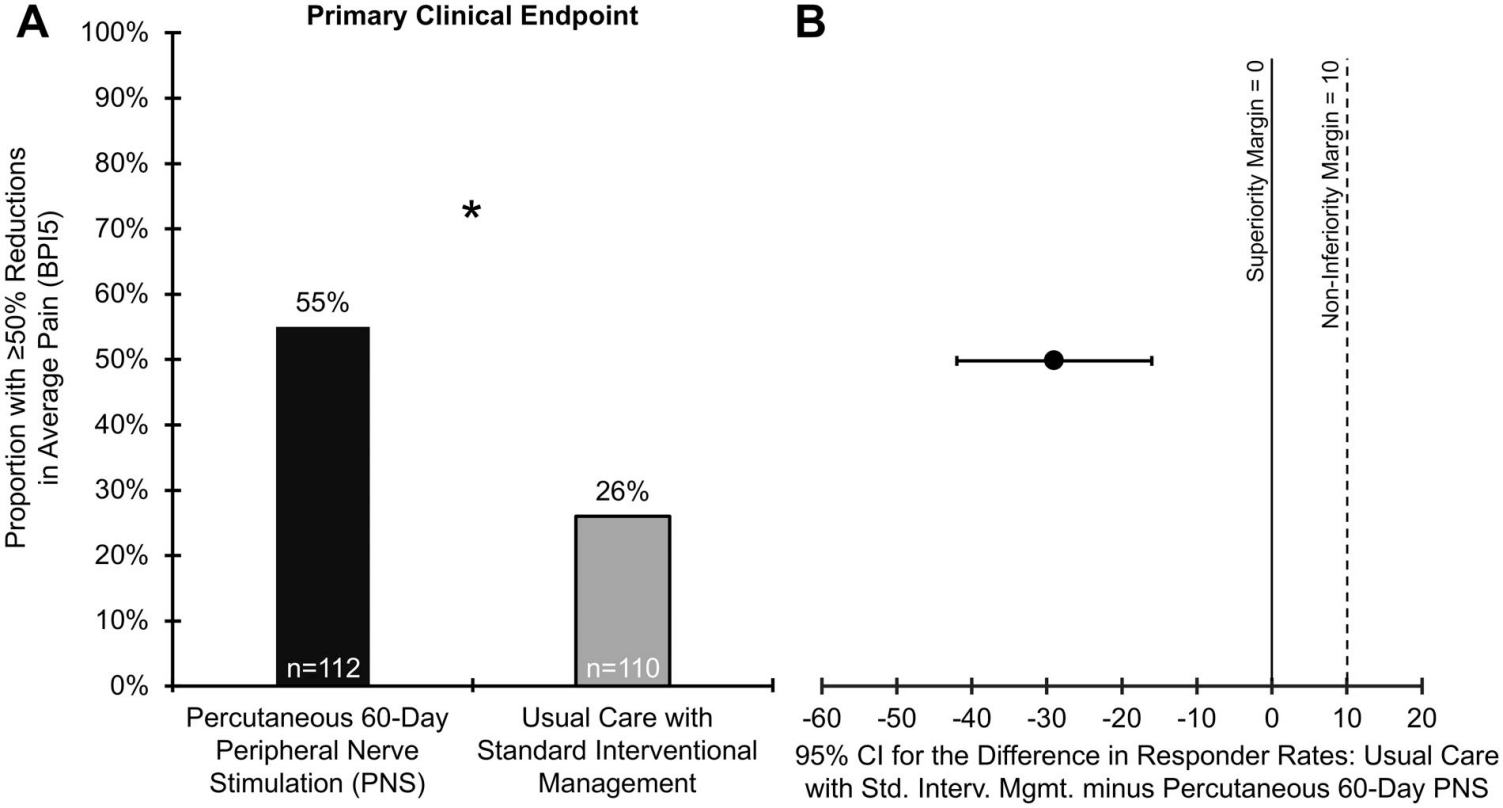
Primary clinical endpoint results: non-inferiority and superiority of the proportion of participants experiencing ≥50% reductions in CLBP with percutaneous 60-day PNS compared to usual care with standard interventional management. (A) Three months post-treatment, the Primary Clinical Endpoint assessed the proportion of participants experiencing ≥50% reductions in average low back pain intensity (BPI5). Missing data were imputed using predictive mean matching and were pooled across 50 datasets. (B) The Primary Clinical Endpoint was analyzed using the Newcombe Hybrid Score Method under a closed procedure of sequential testing for non-inferiority followed by superiority. The 1-sided 97.5% confidence interval (CI) for the differences in proportions (usual care with standard interventional management minus percutaneous 60-day PNS) was less than 10%, the prespecified non-inferiority margin, thus confirming non-inferiority of percutaneous 60-day PNS. Subsequently, the 2-sided 95% CI for the differences in proportions was less than 0%, the prespecified superiority margin, thus confirming superiority of percutaneous 60-day PNS. As such, the proportion estimate of percutaneous 60-day PNS participants who experienced success (55%, *n* = 112) was both non-inferior and superior compared to the proportion estimate of participants who experienced success after receiving usual care with standard interventional treatments (26%, *n* = 110, *P* < .001) for back pain. *P < .05. Abbreviations: BPI5, average pain intensity; BPI, Brief Pain Inventory; CI, confidence interval; PNS, peripheral nerve stimulation; Std. Interv. Mgmt., standard interventional management.

At the 3-month Primary Endpoint, a statistically significantly greater proportion of participants receiving percutaneous 60-day PNS reported ≥50% reductions in average low back pain intensity (55%; *n* = 112, 95% confidence interval [CI] = 45-65) compared to those receiving usual care with standard interventional management (26%; *n* = 110; 95% CI = 17-34; *P* < .001; Figure 3). In accordance with the prospectively defined hypothesis, the proportion estimate for participants with ≥50% reductions in BPI5 for percutaneous 60-day PNS was both statistically non-inferior (non-inferiority margin = 10) and superior compared to that of usual care with standard interventional management (Figure 3).

### Secondary endpoints: additional patient-centric outcomes

**Figure 4.**
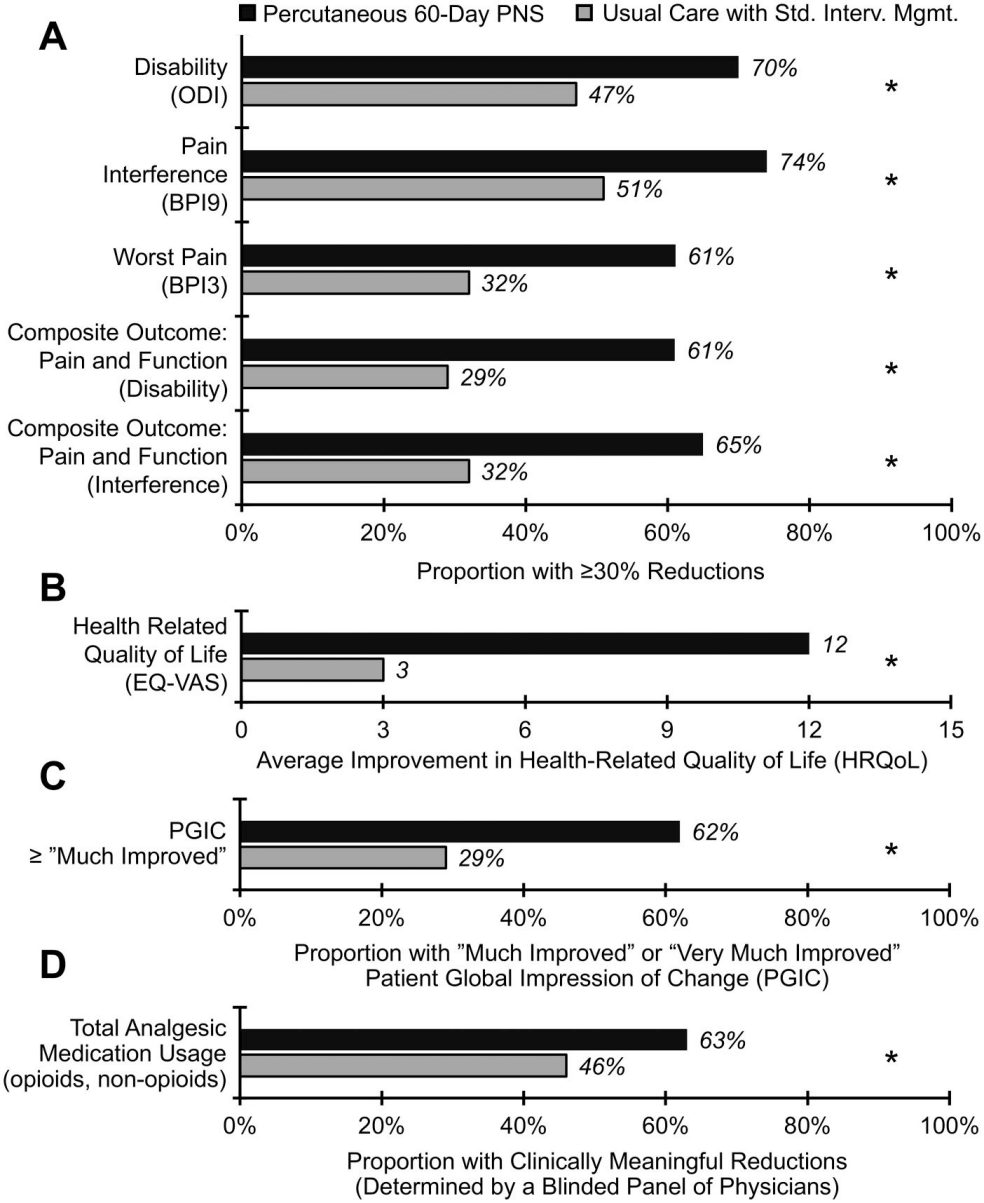
Secondary endpoint results, including impact of treatment on disability, pain interference, composite outcomes, PGIC, and health-related QoL. Results for secondary endpoints are shown at 3 months post-treatment. (A) Compared to usual care with standard interventional management (Group 2; *n* = 110), a greater proportion of participants receiving percutaneous 60-day PNS (Group 1; *n* = 112) reported improvements in back pain-related disability (≥30% ODI; Group 1: 70%, Group 2: 47%), pain interference with daily activities (≥30% BPI9; Group 1: 74%, Group 2: 51%), and worst pain intensity (≥30% BPI3; Group 1: 61%, Group 2: 32%). Composite outcomes included pain and disability (≥30% BPI5 and ODI, Group 1: 61%, Group 2: 29%), and pain and pain interference with daily activities (≥30% BPI5 and BPI9, Group 1: 65%, Group 2: 32%). (B) The percutaneous 60-day PNS group reported greater mean improvements in health-related quality of life (EQ-VAS; Group 1: 12 vs Group 2: 3). (C) A larger proportion of participants receiving percutaneous 60-day PNS compared to usual care with standard interventional management reported their patient global impression of change (PGIC) as either “Much Improved” or “Very Much Improved” (62% vs 29%; *P* < .001). (D) A greater proportion of participants receiving percutaneous 60-day PNS reported clinically meaningful reductions in total analgesic consumption (Group 1: 63%, *n* = 75 vs Group 2: 46%, *n* = 76). *P < .05. Abbreviations: BPI, Brief Pain Inventory; EQ-VAS, EuroQol Visual Analog Scale; HRQol, health-related quality of life; ODI, Oswestry Disability Index; BPI9, pain interference; PGIC, patient global impression of change; PNS, peripheral nerve stimulation; Std. Interv. Mgmt., standard interventional management; BPI3, worst pain.

The main 3-month secondary outcomes are shown in Figure 4 and Table 3, with additional details provided in Appendix S3. For average pain, disability, pain interference with daily activities, and worst pain, a greater proportion of participants in the percutaneous 60-day PNS group (Group 1) demonstrated ≥30% reductions, ≥50% reductions, as well as greater improvements in raw score changes (ie, mean point change) and mean percent change compared to baseline versus participants in the active control group (Group 2) (Table 3, *P* values < .05). Similarly, a greater proportion of 60-day PNS participants reported improvements in composite outcomes requiring each of ≥30% and ≥50% reductions in at least one domain for (1) average pain and/or disability and (2) average pain and/or pain interference. Additionally, a greater proportion of 60-day PNS participants reported improvements in composite outcomes requiring ≥30% reductions in multiple domains for (1) average pain and disability and (2) average pain and pain interference. For patient global impression of change, 60-day PNS participants reported a greater mean improvement in PGIC, and a greater proportion with “Minimally Improved,” “Much Improved,” and “Very Much Improved” PGIC. For health-related quality of life, 60-day PNS participants reported a greater mean improvement and a greater mean percent change than those treated with usual care with standard interventional management.

**Table 3.** Outcomes at 3 months.

	Percutaneous 60-day PNS (Group 1, *n* = 112)		Usual care with standard interventional management (Group 2, *n* = 110)		*P*
***Average pain intensity (BPI5)***					
Mean point change in BPI5	3.1	2.7-3.5	1.5	1.0-2.0	<.001
Mean % change in BPI5	51	44-58	24	16-32	<.001
Proportion with ≥30% BPI5 improvement	75	66-83	41	31-51	<.001
Proportion with ≥50% BPI5 improvement	55	45-65	26	17-34	<.001
***Oswestry Disability Index (ODI)***					
Mean point change in ODI	22	18-25	12	8-16	<.001
Mean % change in ODI	49	41-56	22	13-32	<.001
Proportion with ≥30% ODI improvement	70	61-79	47	36-57	<.001
Proportion with ≥50% ODI improvement	53	43-63	28	19-38	<.001
Proportion with ≥10-pt ODI improvement	74	65-83	55	45-66	.007
Proportion with ≥20-pt ODI improvement	52	42-62	31	22-41	.004
***Pain interference (BPI9)***					
Mean point change in BPI9	3.6	3.0-4.2	1.9	1.3-2.4	<.001
Mean % change in BPI9	57	49-66	27	17-36	<.001
Proportion with ≥30% BPI9 improvement	74	66-83	51	42-61	<.001
Proportion with ≥50% BPI9 improvement	63	53-73	33	24-42	<.001
***Worst pain intensity (BPI3)***					
Mean point change in BPI3	3.4	2.8-3.9	1.3	0.8-1.7	<.001
Mean % change in BPI3	45	37-53	16	10-23	<.001
Proportion with ≥30% BPI3 improvement	61	51-71	32	23-41	<.001
Proportion with ≥50% BPI3 improvement	48	38-58	16	9-23	<.001
***Patient global impression of change (PGIC)***					
Mean PGIC score	1.8	1.5-2.0	0.7	0.4-0.9	<.001
Proportion with ≥1 minimally improved PGIC	87	79-92	56	46-66	<.001
Proportion with ≥2 much improved PGIC	62	53-72	29	20-37	<.001
Proportion with ≥3 very much improved PGIC	33	23-43	5	1-10	<.001
***Composite outcomes***					
Proportion with ≥30% BPI5 and/or ≥30% ODI	84	77-91	59	49-70	<.001
Proportion with ≥30% BPI5 and ≥30% ODI	61	51-71	29	20-37	<.001
Proportion with ≥50% BPI5 and/or ≥50% ODI	69	60-78	39	28-49	<.001
Proportion with ≥30% BPI5 and/or ≥30% BPI9	84	77-91	61	51-70	<.001
Proportion with ≥30% BPI5 and ≥30% BPI9	65	55-74	32	23-41	<.001
Proportion with ≥50% BPI5 and/or ≥50% BPI9	70	61-79	41	31-50	<.001
***EQ-VAS***					
Mean point change in EQ-VAS	12	6-17	3	−2 to 8	.022
Mean % change in EQ-VAS	30	17-44	13	4-22	.035

Statistical results evaluated at 3 months post-treatment. Means and proportions are shown with [95% confidence intervals] for each endpoint. Missing data were imputed using predictive mean matching and were pooled across 50 datasets.

Abbreviations: BPI, Brief Pain Inventory; BPI3, worst pain intensity; BPI5, average pain intensity; BPI9, pain interference; EQ-VAS, EuroQol Visual Analog Scale; ODI, Oswestry Disability Index; PGIC, patient global impression of change; PNS, peripheral nerve stimulation; Outcomes with continuous data (ie, point change or % change): a 2-way Wilcoxon rank-sum test; Outcomes with proportions (%): testing differences between 2 proportions with 95% confidence intervals; 95% CI, 95% confidence interval.

Results from the blinded physician panel regarding analgesic consumption are shown in Figure 4 and Table S2. In Group 1, 63% (*n* = 47/75) of participants reported a clinically meaningful reduction in analgesic use (including opioid and non-opioid medication) compared to 46% (*n* = 35/76) of Group 2 participants (*P* = .018) at 3 months post-treatment. For opioid reduction, 64% (*n* = 9/14) of those who received 60-day PNS were able to meaningfully reduce intake versus 21% who received usual care with standard interventional management (*n* = 3/14, *P* = .054).

Outcomes at 3 months as observed, without imputation for missing data, are shown in Table S3. Results from the prespecified subgroup analyses comparing outcomes following percutaneous 60-day PNS with those among participants who received specific CLBP interventions (ie, excluding participants who declined intervention) are shown in Table S4.

### Durability of relief

**Figure 5.**
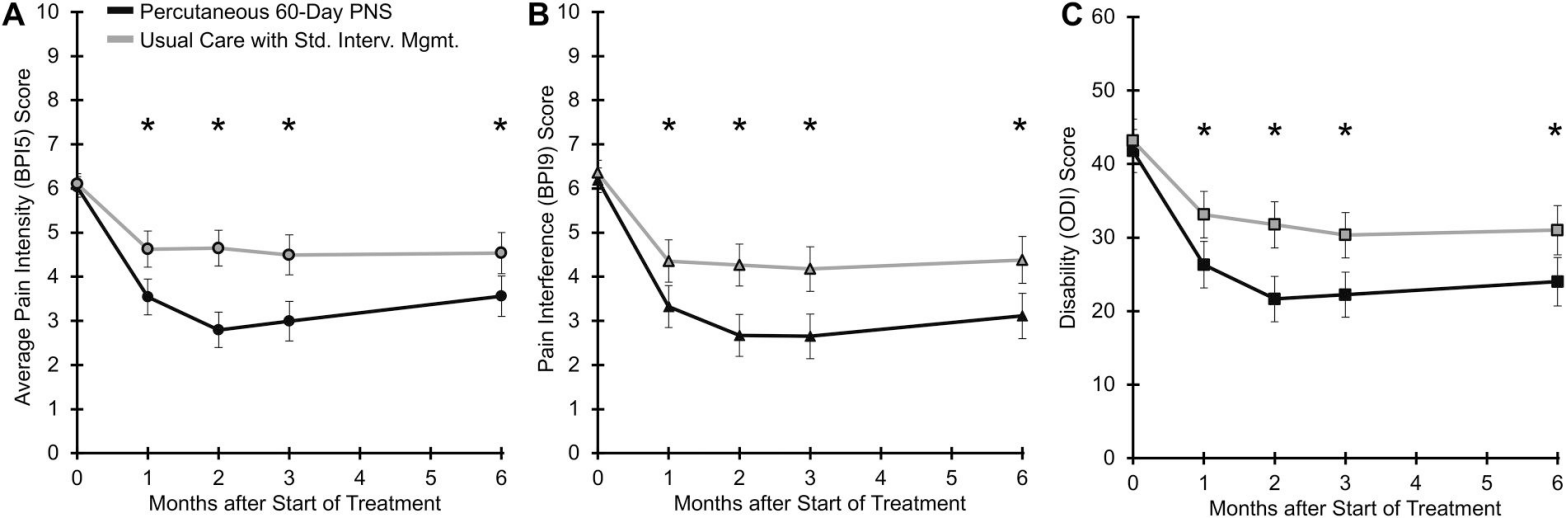
Long-term improvements in CLBP with percutaneous 60-day PNS compared to usual care with standard interventional management. The reductions in pain intensity (BPI5), pain interference (BPI9), and disability (ODI) were greater for all completed timepoints with percutaneous 60-day PNS (Group 1) compared to usual care with standard interventional management (Group 2). The least square means [95% confidence intervals] for all participants in each group are shown for (A) pain intensity, (B) interference, and (C) disability over time. * *P* < .05. Abbreviations: BPI5, average pain intensity; BPI, Brief Pain Inventory; mo, month; BPI9, pain interference; PNS, Peripheral Nerve Stimulation; Std. Interv. Mgmt., standard interventional management.

As shown in Figure 5 and Table 2, through 6 months post-treatment, those treated with percutaneous 60-day PNS reported greater reductions in CLBP average intensity (BPI5, Figure 5A; *P* values < .001), pain interference (BPI9, Figure 5B; *P* values ≤ .003), and disability (ODI, Figure 5C; *P* values ≤ .004) than those receiving usual care with standard interventional management.

### Adverse events

A total of 111 participants in Group 1 and 102 participants in Group 2 underwent a medical procedure as part of this clinical trial and were included in the Safety Analysis Set. No serious, unanticipated study-related adverse events occurred in any participants. As shown in Table S5, the most common adverse events (AEs) related to the use of percutaneous 60-day PNS were consistent with previously published trials and were most commonly associated with the waterproof dressing and skin adhesive products, such as mild skin irritation (35 participants; 31.5%) or itching at the dressing or mounting pad (22 participants; 19.8%). Other less common AEs related to percutaneous 60-day PNS included pain (9 participants; 8.1%), discomfort (6 participants; 5.4%), discoloration (1 participant, 0.9%), Other: neurological/other (eg, muscle spasms, stinging sensations; 4 participants, 3.6%), and Other: cardiovascular (eg, temporary vasovagal response; 1 participant, 0.9%). Additionally, there were 6 reports of superficial skin infections (2.7% rate of occurrence per lead exit site) and 3 reports of granulomas (1.4% rate of occurrence per lead exit site). The most common adverse events related to usual care with standard interventional management were new or worsening of pain following an intervention. All study adverse events were non-serious (mild or moderate) and were followed to resolution.

## Discussion

This pragmatic prospective multicenter RCT met its Primary Clinical Endpoint, with a greater proportion of participants treated with percutaneous 60-day PNS reporting ≥50% reductions in average back pain intensity at 3 months post-treatment compared to usual care with standard interventional management. Results from the Primary Clinical Endpoint were supported by consistent findings across secondary, patient-centric outcomes of disability, pain interference, quality of life, and analgesic consumption. This RCT provides compelling evidence supporting the therapeutic potential of percutaneous 60-day PNS in the management of CLBP.

The goal of this RCT was to evaluate the effectiveness of percutaneous 60-day PNS compared to an active control of usual care with standard interventions for CLBP. The treatment algorithms for CLBP are physician dependent and evolve over time to meet the needs and diagnoses of the patients, consistent with personalized medicine. Despite there being no restriction on the type or frequency of intervention in the active control group, several commonly recommended treatments were used infrequently or not at all (eg, basivertebral nerve ablation, spinal cord stimulation, surgery) in the time period before the Primary Endpoint. Nonetheless, the treatment utilization among the active control group (ie, physicians most commonly recommended ablations, injections, and physical therapy) was generally consistent with clinical practice. The complex nature of CLBP is reflected in the range of treatments and success rates for the active control group (eg, 26% with ≥50% pain relief in the active control group). Participants in this study were required to have failed at least 2 prior treatments. The variation in those treatments created a heterogenous participant population that could have received a treatment in the study that they already failed. An analysis of historical treatment outcomes data found that among the 36 Group 2 participants who received RFA as an intervention in this study and went on to complete the Primary Endpoint, only 4 reported prior use and failure of that same treatment prior to enrollment. Literature shows that the specific interventions used in the control group can be effective in well-selected patients, especially in more homogenized treatment regimes, and are broadly consistent with findings from published studies and meta-analyses.^14–17^ The present findings reflect the challenge of treating a heterogeneous and recalcitrant population with moderate to severe pain. They do not necessarily imply a lack of efficacy in usual care with standard interventional management, as the active control group showed improvement in a number of outcomes.

The results from this RCT suggest benefits from percutaneous 60-day PNS may, through pain relief, extend to health domains beyond just pain. Here, participants treated with percutaneous 60-day PNS reported greater improvements in average pain, back pain-related disability, pain interference with daily activities, worst pain intensity, health-related quality of life, and patient global impression of change. Initial pain relief from 60-day PNS may be due to a stimulation pathway that directly activates large-diameter (sensory) afferent nerve fibers (proprioceptive Aα/β nerve fibers) and directly activates efferent (motor) nerve fibers, which elicits further indirect activation of proprioceptive sensory Aα/β nerve fibers (eg, via cyclic muscle tension). Together, these activations are believed to engage central mechanisms of pain relief (eg, gate control mechanism of pain relief).^8^ Although more research is needed to establish long-term durability beyond 6 months, improvements in pain, pain interference, and disability through 6 months with 60-day PNS, are consistent with prior studies^7^^,^^9^^,^^18^^,^^19^ and may be due to several factors. One theory for prolonged pain relief following the discontinuation of stimulation and subsequent lead removal involves peripherally induced modulation of central sensitization,^8^ where stimulation-evoked signaling in the periphery may provide pain relief and increase normal, healthy neural signaling, as experimental evidence suggests that proprioceptive nerve signaling can influence maladaptive central neuroplasticity.^20^^,^^21^ The theory suggests that the combination of pain relief plus increased healthy neural signaling may reverse maladaptive pain processing, enabling patients to resume normal daily activities (eg, exercise and sleep) with less pain, which in turn helps to further perpetuate pain relief beyond the 60-day stimulation period. Further research is needed to validate the theorized mechanism of action (MOA), and the findings from this RCT merit deeper exploration. The magnitude of the reductions in pain observed with percutaneous 60-day PNS in the present RCT (mean 3.1-point reduction) are relatively consistent with a review of the findings of a recent meta-analysis evaluating PNS of the medial branch nerves across multiple studies and approaches,^22^ despite differences in device design, stimulation parameters, treatment duration, time course of pain relief, theorized MOA, technique, and other variables (eg, etiologies, applications, etc).

A computational model of health economic outcomes data suggests that costs associated with CLBP treatment can be reduced when percutaneous 60-day PNS is introduced early in the treatment continuum, since the treatment may reduce the need for diagnostic testing or sequential interventional treatments.^23^ The pragmatic design of the present study provides perspective on the clinical utility of a percutaneous 60-day PNS treatment and complements the existing literature by providing a real-world comparison to usual care including standard interventional management for CLBP.

As with many comparative-effectiveness studies, this research has limitations. The pragmatic study design has the benefits of generalizability but also reduces experimental control. As an example, blinding was not possible following randomization and thus can introduce potential sources of bias (eg, detection bias, placebo effects). Patients in the PNS group may have had higher expectations^24^ (eg, expectation bias, Hawthorne effect) and these differences may diminish equipoise between treatment groups, which could influence outcomes. In Group 1, the initial treatment (60-day PNS) was paid for by the study. In Group 2, interventions were based on recommendations from physician investigators; yet costs were covered by individual’s insurance, not by the study, and some participants may have received recommendations for interventions (eg, surgery, spinal cord stimulation) but opted not to pursue them (eg, due to insurance denials, other cost considerations, perceived risk, or invasiveness). This difference could lead to biases between groups (eg, performance bias and/or selection bias). Additionally, non-specific effects could also influence the study’s observations. Since the most common treatments for Group 2 were ablations and therapeutic injections, non-specific effects are likely comparable across treatment groups, with most participants in both groups receiving needle-based interventional procedures as part of the study. Whereas prior studies have demonstrated the efficacy of percutaneous 60-day PNS versus sham stimulation in other pain conditions,^5–7^ the absence of a placebo control is a limitation of this study. However, as previously noted, this study was designed with the goal of generating clinically relevant data on the comparative effectiveness of 60-day PNS versus real-world care. Finally, this study was conducted during the COVID-19 pandemic which adversely and unexpectedly affected administrative processing, clinical site operations, and subject participation and compliance.

## Conclusions

This prospective, multicenter pragmatic randomized controlled trial met its Primary Clinical Endpoint, with a greater proportion of participants treated with percutaneous 60-day PNS reporting ≥50% reductions in average back pain intensity 3 months post-treatment compared to usual care with standard interventional management (eg, physical therapy, ablations, injections). Those treated with percutaneous 60-day PNS reported greater improvements in disability, pain interference, health-related quality of life, and analgesic consumption. Although prospective follow-up remains ongoing while participants continue participation beyond the completed 6-month visit, significant reductions in pain and the resulting improvements in function were sustained through 6 months with percutaneous 60-day PNS. Collectively, the results from this study complement existing literature and support the use of percutaneous 60-day PNS as a treatment option for CLBP.

## Supplementary Material

pnaf147_Supplementary_Data

## Data Availability

The datasets generated during and/or analyzed during the current study are available from the corresponding author on reasonable request.

## Thank you to participants

The authors extend sincere appreciation to the patients who participated in this study for their time and valuable contributions; your involvement made this work possible.
