# Supplementary material for: Comparison of percutaneous 60-day peripheral nerve stimulation of the lumbar medial branches to usual care with standard interventional management for chronic low back pain—a multicenter pragmatic randomized controlled trial (RESET)
Source: Pain Med. 2025 Oct 25;27(4):462–73. doi: 10.1093/pm/pnaf147 (PMC13061121; doi:10.1093/pm/pnaf147)
Supplement: pnaf147_Supplementary_Data [file pnaf147_supplementary_data.docx]

**Comparison of Percutaneous 60-Day Peripheral Nerve Stimulation of the Lumbar Medial Branches to Usual Care with Standard Interventional Management for Chronic Low Back Pain – a Multicenter Pragmatic Randomized Controlled Trial (RESET) McCormick et al.**

**APPENDIX 1 - Study Eligibility Criteria**

Inclusion Criteria

- Age ≥21 years and ≤75 years
- Able to understand and comply with study requirements and provide written informed consent
- Chronic low back pain (*i.e.,* pain lasting longer than 6 months, where the vast majority and center of pain is in the lumbar region, L1-L5 vertebral levels)
- Reports an average low back pain score ≥ 4 on a scale of 0-10 (BPI5)
- Previous use of at least two types of LBP therapies (e.g., medications, physical therapy, injections, etc.)
- At least 4 weeks of stable pain treatment or medication as indicated by subject reported medication history (*i.e.,* no new treatments or change in medication for pain in the last 4 weeks)
- Reports active health insurance coverage (*e.g.,* through commercial insurance, Medicare, Medicaid, Tricare)

Exclusion Criteria

- Radicular leg pain (*e.g.,* pain that spreads to the lower extremities) or referred pain outside the lumbar region (*e.g.,* sacral pain, hip pain) that is ≥4 on a scale of 0-10, BPI5
- Pain in the thoracic or cervical region that is ≥4 on a scale of 0-10, BPI5
- Pain from the sacroiliac joint, as determined by the Investigator
- Signs of infection on or around the low back, or other conditions that increase risk to the subject in the opinion of the Investigator (*e.g.,* valvular heart disease that creates an infection risk, compromised immune system, history of recurrent skin infections)
- Signs of serious underlying cause of low back pain as determined by the Investigator, (*e.g.,* cancer, chronic infection, referred visceral pain, metabolic bone disorder)
- Deep brain stimulation (DBS) system, an implanted active cardiac implant, or any other implantable neurostimulator whose stimulus current pathway may overlap the Percutaneous 60-Day PNS stimulator’s current pathway
- Anesthetic or corticosteroid injections in the low back (including trigger point, epidural, intrathecal, facet, or sacroiliac joint injections) within the last 3 months (not including a diagnostic medial branch block), or botulinum toxin (Botox) injection in the low back within the last 6 months
- Prior radiofrequency ablation of the lumbar medial branches (L1-L5) within the last 6 months
- Prior lumbar surgery
- Clinically relevant stenosis of the central canal or foramina as determined by the Investigator (*e.g.,* symptoms of neurogenic claudication)
- Greater than mild lumbar scoliosis (*i.e.,* >20 degrees)
- Condition that could impact response to percutaneous PNS (*e.g.,* fibromyalgia confirmed by a specialist, multiple sclerosis, spinal cord injury)
- History of significant trauma to the lumbar spine or paraspinal musculature (*e.g.,* burst fractures or fracture dislocations)
- Score of >20 on the Beck Depression Inventory (BDI-II)
- Average Pain Interference score <4 using BPI9
- Obese with a body mass index (BMI) >40
- Current illicit substance abuse or high-dose opioid dependence (e.g., daily opioid usage ≥90 mg morphine equivalents)
- Current participation, or less than 30 days from completing any drug or device trial, or previously received Percutaneous 60-Day PNS for LBP
- Pending litigation, workers compensation or other secondary gain issues
- Tape or adhesive allergy
- Allergy to all local anesthetic agents (*e.g.,* lidocaine)
- Any other medical condition that may interfere with ability to participate in a clinical trial as determined by the Investigator (*e.g.,* bipolar disorder)
- Vulnerable populations (*e.g.,* prisoners, individuals that report to investigators)
- Bleeding disorder (e.g., hemophilia)
  - Note: anticoagulant use is not considered a bleeding disorder.

Additional Inclusion Criteria *(assessed before randomization)*

- Average pain intensity score of ≥ 4 (determined by calculating the mean “average pain” collected in a 7-day baseline diary, using Question #5 on the BPI-SF)

Additional Exclusion Criteria *(assessed before start of treatment with Percutaneous 60-Day PNS)*

- Pregnancy (Group #1 only)

**Comparison of Percutaneous 60-Day Peripheral Nerve Stimulation of the Lumbar Medial Branches to Usual Care with Standard Interventional Management for Chronic Low Back Pain – a Multicenter Pragmatic Randomized Controlled Trial (RESET) McCormick et al.**

**APPENDIX 2 – Supplementary Text**

**METHODS**

**Recruitment and Assessment**

Recruitment (e.g., referral, in-clinic recruitment, self-selection, advertisements) and screening was conducted at a wide variety of centers (e.g., large academic centers, research institutions, and private practice clinics) in settings with rural and urban populations. All study assessments and outcomes were conducted by trained study personnel (e.g., physician, nurse, or study coordinator). At baseline, CLBP-related history (*e.g.,* prior treatments, imaging results) was obtained and participants completed a pain diagram and a physical exam to confirm eligibility (e.g., confirm the majority of pain was from the lumbar region).

**Primary Safety Endpoint**

The Primary Safety Endpoint was prospectively-defined as the occurrence and type of study-related adverse events (AEs). AEs for Group #1 were collected through a combination of open query and direct query (e.g., “Does the subject have any signs or symptoms of infection or inflammation, such as redness, pain, or swelling at the [PNS] lead exit site(s)?”) reporting. AE collection for Group #2 relied on open query reporting, as AEs could vary for different interventions received as part of usual care. For each AE, investigators determined its relation to the study device or procedures and categorized the severity as “mild” if they caused inconvenience and resolved without treatment, “moderate” if they required medical treatment or intervention, or “severe” if they required medical treatment or intervention to prevent permanent damage, required hospitalization, or resulted in permanent damage or death. The Safety Analysis Set included all participants who underwent a study procedure (*e.g.,* lead placement, change in medication, medial branch block, diagnostic procedure, or standard interventional treatment), excluding participants who dropped out prior to receiving any diagnostic or treatment. An Independent Medical Reviewer adjudicated each AE as “nonserious” or “serious”.

**Statistical Methods**

A power analysis using previously published success rates for both the 60-day PNS group and the usual care with standard interventional management group determined that a sample size of 230 enrolled participants (approximately 115 per group) would be sufficient to assess the Primary Clinical Endpoint with ≥80% power (α=0.05), while accounting for attrition and drop-out of approximately 20%. In the event of missing diary scores for average pain intensity (e.g., if a participant recorded 5 of 7 days of their BPI5 Diary leading up to the clinic visit), the prospectively defined statistical analysis plan specified that missing scores be replaced using BPI5-Recall (*i.e.,* a single score “recall” of their average pain over the past week) collected during the clinic visit, when available. The same replacement process was used for BPI3 Diaries, when BPI3-Recall scores were available. For all outcome measures, statistical data analyses are reported with imputation for missing data where appropriate.

**RESULTS**

**Study Population and Baseline Demographics**

Across 21 recruitment sites, an average of 11.1 ± 10.3 participants were enrolled (median: 7 participants; interquartile range: 12). Prospective follow-up remains ongoing while participants continue participation beyond the completed 6-month visit. Among the 222 participants in the Full Analysis Set, 4 identified their race as American Indian or Alaskan Native, 5 as Asian, 50 as Black or African American, 0 as Native Hawaiian or other Pacific Islander, and 167 as White. A total of 4 participants identified as more than 1 race. Regarding ethnicity, 8 participants identified as Hispanic. The mean participant age was 53 ± 14 years and 52% of participants (n=115/222) were female. The mean duration of CLBP at the time of study screening was 14 ± 13 years. A substantial proportion of participants were reported as having facetogenic (*i.e.,* lumbar spondylosis; n=125/222) or discogenic (*i.e.,* degenerative disc disease; n=84/222) pain, as judged by their enrolling physicians. At baseline, participants rated their CLBP as moderate-to-severe with an average pain intensity (BPI5) of 6.1 ± 1.2. Consistent with high pain scores, participants also reported poor function at baseline (*i.e.,* back pain-related disability and interference of pain on activities of daily living). On average, back pain-related disability was moderate-to-severe at baseline, with an average ODI score of 43 ± 16. Average pain interference with daily activities (BPI9) at baseline was 6.3 ± 1.5. For those in Group #1 with available data at 2 months (i.e., the end of 60-day PNS treatment), the mean stimulation usage reported was 7.7 ± 2.3 hours per day (n=89).

**Secondary Endpoints: Additional Patient-Centric Measures of Function and Quality of Life**

Participants in the percutaneous 60-day PNS group reported a greater mean reduction in average pain intensity compared to the usual care with standard interventional management group at the Primary Endpoint (3.1-pt vs. 1.5-pt reduction, p<0.001; 51% vs. 24% reduction, p<0.001; **Table 3**). An additional analysis revealed that a greater proportion of participants receiving percutaneous 60-day PNS experienced ≥30% improvement in BPI5 compared to usual care with standard interventional management (75% vs. 41%, p<0.001; **Table 3**).

For the functional endpoint of back pain-related disability (ODI), percutaneous 60-day PNS resulted in larger mean point reductions (22-pt vs. 12-pt, p<0.001) and mean percent changes from baseline (49% vs. 22%, p<0.001) compared to usual care with standard interventional management. A greater proportion of participants receiving percutaneous 60-day PNS experienced ≥30% reductions in disability compared to usual care with standard interventional management (70% vs. 47%, p<0.001; **Table 3**, **Fig. 4**). A greater proportion of participants receiving percutaneous 60-day PNS were responders when thresholds for success were set at either ≥50% ODI improvement or ≥20-pt ODI improvement (**Table 3;** p-values<0.05).

For the functional endpoint of pain interference with daily activities (BPI9), percutaneous 60-day PNS also resulted in larger mean point reductions (3.6-pt vs. 1.9-pt, p<0.001) and mean percent changes from baseline (57% vs. 27%, p<0.001) compared to usual care with standard interventional management. A larger proportion of participants receiving percutaneous 60-day PNS experienced ≥30% improvements in pain interference compared to usual care with standard interventional management (74% vs. 51%, p<0.001; **Table 3**, **Fig. 4**).

For worst pain intensity (BPI3), percutaneous 60-day PNS provided larger point reductions (3.4-pt vs. 1.3-pt, p<0.001) and mean percent change from baseline compared to usual care with standard interventional management (45% vs. 16%, p<0.001). A greater proportion of percutaneous 60-day PNS participants reported a ≥30% reduction in BPI3 (61% vs. 32%, p<0.001; **Table 3**, **Fig. 4**).

At 3 months post-treatment, participants in Group #1 receiving percutaneous 60-day PNS reported greater improvement in their quality of life compared to Group #2 usual care with standard interventional management, calculated as a greater mean PGIC score (1.8 vs. 0.7, on scale of -3 to +3, p<0.001; **Table 3**). When considering the proportion of participants reporting PGIC as either “Much Improved” (+2) or “Very Much Improved” (+3), a larger proportion of participants receiving percutaneous 60-day PNS compared to usual care with standard interventional management reported benefit (62% vs. 29%; p<0.001, **Fig. 4**). More participants receiving percutaneous 60-day PNS reported the highest possible level of improvement in quality of life (“Very Much Improved”, 33%), compared to 5% of participants receiving usual care with standard interventional management (p<0.001).

Health-related quality of life was assessed using EQ-5D-5L visual analog scale (EQ-VAS; from 0-100, with 100 representing the best imaginable health and 0 representing the worst). Participants receiving percutaneous 60-day PNS reported greater mean point improvements from baseline compared to usual care with standard interventional management (12-pt vs. 3-pt change, p=0.022; **Table 3**, **Fig. 4**). Percutaneous 60-day PNS participants reported larger mean percent improvements in health-related quality of life than usual care with standard interventional management (30% vs. 13%, p=0.035; **Table 3**).

**Composite Endpoints**

For a composite endpoint requiring improvements in both average back pain intensity and back pain-related disability (*i.e.,* ≥30% BPI5 and ≥30% ODI), a larger proportion of participants receiving percutaneous 60-day PNS experienced improvements compared to usual care with standard interventional management (61% vs. 29%; p<0.001; **Table 3**, **Fig. 4**). A greater proportion of participants also reported improvements for another composite endpoint, which required improvement in both average back pain intensity and a functional endpoint of pain interference (*i.e.,* ≥30% BPI5 and ≥30% BPI9) compared to usual care with standard interventional management (65% vs. 32%; p<0.001; **Table 3**, **Fig. 4**). Other variations of composite endpoints are reported in **Table 3**.

**Medication Usage**

As shown in **Supplementary Table S2** and **Fig. 4** a review of participants’ weekly medication diaries at baseline and 3 months post-treatment by a blinded panel of physicians found that 63% (n=47/75) of Group #1 participants reported a clinically meaningful reduction in total medication usage compared to 46% (n=35/76) of Group #2 participants (p=0.018). Considering all non-opioid analgesic medications used for back pain, the blinded physician panel found that 69% (n=46/67) of Group #1 participants had a clinically meaningful reduction compared to 50% (n=35/70) of Group #2 participants (p=0.023). Among those with data at Primary Endpoint, 15% (n=14/91) assigned to Group #1 and 15% (n=13/88) assigned to Group #2 reported taking opioids for CLBP at baseline. On average, the daily opioid consumption at baseline was 26 morphine milligram equivalents (MME) for both Groups #1 and #2. One additional participant in Group #2 started taking opioids during treatment. The blinded panel found that 64% (n=9/14) of Group #1 participants compared to 21% (n=3/14) in Group #2 had a clinically meaningful reduction in total opioid analgesic usage (p=0.054). An analysis of changes in daily opioid MME at 3 months post-treatment found that 50% of participants in Group #1 (n=7/14) and 21% in Group #2 (n=3/14) reported ≥50% reductions in daily opioid analgesic consumption (p=0.237).

**Primary Clinical Endpoint Results: Observed Dataset**

The findings from the imputed dataset were consistent with those from the observed dataset, where a greater proportion of participants (56%; n=90, 95% CI = [45, 66]) receiving percutaneous 60-day PNS reported ≥50% reductions in average low back pain intensity (BPI5) compared to those receiving usual care with standard interventional management (26%; n=88 participants; 95% CI = [17, 37]; p<0.001; **Supplementary Table S3**). Additional scenario-based analyses validate the robustness of study conclusions at the Primary Clinical Endpoint, with worst-case scenarios (Group #1: 45% vs. Group #2: 21%; p<0.001) and best-case scenarios (Group #1: 64% vs. Group #2: 41%; p<0.001) demonstrating significant differences between groups.

**Group #1 and Group #2 Treatment Subgroups**

The study’s statistical analysis plan included prespecified subgroup analyses to compare percutaneous 60-day PNS to key standard interventions enabling a more precise assessment of their relative effectiveness and to account for the influence of participants who opted not to receive an intervention. **Supplementary Table S4** shows results for the study’s Primary Clinical Endpoint (*i.e.,* the proportion of participants experiencing ≥50% reductions in BPI5 at 3 months post-treatment) among participants in Group #2 who received treatments for CLBP. The proportion reporting 50% reductions in BPI5 with percutaneous 60-day PNS (56%, n=90) was greater than those treated with RFA (26%, n=38; p=0.003), therapeutic injections (26%, n=34; p=0.005), and conservative care (e.g., used only PT, chiropractic treatment, TENS, or massage, etc.; 13%, n=8, p=0.026).

**Comparison of Percutaneous 60-Day Peripheral Nerve Stimulation of the Lumbar Medial Branches to Usual Care with Standard Interventional Management for Chronic Low Back Pain – a Multicenter Pragmatic Randomized Controlled Trial (RESET) McCormick et al.**

**APPENDIX 3 - Supplementary Tables**

| **S U P P L E M E N T A R Y T A B L E S1.** Diagnostic Procedures and Interventions Used by Group #2 Prior to Primary Endpoint | |
| --- | --- |
| ***Diagnostic Procedures*** | |
| Lumbar Medial Branch Block (MBB), *n participants (n procedures)* | 53 (78) |
| Diagnostic Imaging (MRI, X-ray, etc.)*, n participants (n procedures)* | 28 (37) |
| Other (e.g., Discogram, Provocative Discography, SCS Trial)*, n participants (n procedures)* | 7 (8) |
| ***CLBP Interventions*** | |
| Lumbar Radiofrequency Ablation (RFA), *n participants (n procedures)* | 40 (46) |
| Therapeutic Injections (ESI, Trigger Point, facet, SI, etc.), *n participants (n procedures)* | 36 (51) |
| ***Conservative Care or Other CLBP Treatments*** |  |
| Physical Therapy*, n participants* | 16 |
| Chiropractic Treatment or Spinal Manipulation*, n participants* | 7 |
| Other (e.g., Acupuncture, TENS, Massage Therapy, etc.)*, n participants* | 12 |
| Continuing Usual Care (*i.e.,* no new treatments)*, n participants* | 9 |
| *CLBP-related healthcare utilization shown for Group #2 (Usual Care with Standard Interventional Management, Full Analysis Set) who completed Start of Treatment (n=97). Some participants used more than one diagnostic and/or intervention. Abbreviations: ESI, epidural steroid injection; MBB, medial branch block; MRI, magnetic resonance imaging; RFA, radiofrequency ablation; SI, sacroiliac joint; TENS, transcutaneous electrical nerve stimulation.* | |

| **S U P P L E M E N T A R Y T A B L E S2.** Medication Usage at 3 Months | | | | | | | |  | |
| --- | --- | --- | --- | --- | --- | --- | --- | --- | --- |
|  | Percutaneous 60-Day PNS  (Group #1) | | |  | Usual Care with Standard Interventional Management  (Group #2) | | |  | p-value |
|  |  |  |  |  |  |  |  |  |  |
| ***Assessment of Analgesic Consumption by Blinded Physician Review Panel*** | ***Decrease % (n/N)*** | ***No Change % (n/N)*** | ***Increase % (n/N)*** |  | ***Decrease % (n/N)*** | ***No Change % (n/N)*** | ***Increase % (n/N)*** |  |  |
| Any Medications Impacting Pain  (*i.e.,* opioids and/or non-opioids) | 63 (47/75) | 28 (21/75) | 9.3 (7/75) |  | 46 (35/76) | 28 (21/76) | 26 (20/76) |  | 0.018 |
| Non-opioid Analgesic Consumption Only | 69 (46/67) | 22 (15/67) | 8.9 (6/67) |  | 50 (35/70) | 24 (17/70) | 26 (18/70) |  | 0.023 |
| Opioid Analgesic Consumption Only | 64 (9/14) | 29 (4/14) | 7.1 (1/14) |  | 21 (3/14) | 71 (10/14) | 7.1 (1/14) |  | 0.054 |
| ***Calculated Changes in Opioid Consumption*** |  | | |  |  | | |  |  |
| % Change in MME | 34 (99) | | |  | 25 (42) | | |  | 0.209 |
| ≥ 50% MME Improvement | 50 (7/14) | | |  | 21 (3/14) | | |  | 0.237 |
| *Outcomes with from the blinded Physician Review Panel (%, n/N): a Mantel-Haenszel Chi-Square test compared the ordered distributional responses; Outcomes with continuous data (i.e., % change; mean, SD): a two-way Wilcoxon rank-sum test; Outcomes with proportions (%, n/N): two-sided Fisher’s exact test. Abbreviations: MME, milligrams morphine equivalent; PNS, peripheral nerve stimulation;* | | | | | | | | | |

| **S U P P L E M E N T A R Y T A B L E S3. Outcomes at 3 Months as Observed** |
| --- |

|  | **Observed Dataset** | | | | |
| --- | --- | --- | --- | --- | --- |
|  | Percutaneous 60-Day PNS (Group #1) |  | Usual Care with Standard Interventional Management (Group #2) |  | p-value |
| ***Average Pain Intensity (BPI5)*** | | | |  |  |
| Mean Point Change in BPI5 | 3.1 ± 2.2 |  | 1.6 ± 2.1 |  | <0.001 |
| Mean % Change in BPI5 | 52 ± 35.3 |  | 26 ± 33.8 |  | <0.001 |
| Proportion with ≥30% BPI5 Improvement | 76 (68/90) |  | 44 (39/88) |  | <0.001 |
| Proportion with ≥50% BPI5 Improvement | 56 (50/90) |  | 26 (23/88) |  | <0.001 |
| ***Oswestry Disability Index (ODI)*** | | | |  |  |
| Mean Point Change in ODI | 19 ± 17 |  | 13 ± 16 |  | 0.011 |
| Mean % Change in ODI | 45 ± 37 |  | 27 ± 34 |  | 0.001 |
| Proportion with ≥30% ODI Improvement | 67 (60/90) |  | 47 (40/85) |  | 0.01 |
| Proportion with ≥50% ODI Improvement | 48 (43/90) |  | 28 (24/85) |  | 0.009 |
| Proportion with ≥10-pt ODI Improvement | 71 (64/90) |  | 56 (48/85) |  | 0.058 |
| Proportion with ≥20-pt ODI Improvement | 48 (43/90) |  | 32 (27/85) |  | 0.032 |
| ***Pain Interference (BPI9)*** | | | |  |  |
| Mean Point Change in BPI9 | 3.6 ± 2.7 |  | 2.2 ± 2.4 |  | <0.001 |
| Mean % Change in BPI9 | 58 ± 40 |  | 34 ± 41 |  | <0.001 |
| Proportion with ≥30% BPI9 Improvement | 76 (68/89) |  | 56 (49/87) |  | 0.007 |
| Proportion with ≥50% BPI9 Improvement | 64 (57/89) |  | 38 (33/87) |  | <0.001 |
| ***Worst Pain Intensity (BPI3)*** | | | |  |  |
| Mean Point Change in BPI3 | 3.5 ± 2.7 |  | 1.6 ± 2.3 |  | <0.001 |
| Mean % Change in BPI3 | 47 ± 35 |  | 21 ± 33 |  | <0.001 |
| Proportion with ≥30% BPI3 Improvement | 61 (55/90) |  | 36 (32/88) |  | 0.001 |
| Proportion with ≥50% BPI3 Improvement | 49 (44/90) |  | 19 (17/88) |  | <0.001 |
| ***Patient Global Impression of Change (PGIC)*** | | | |  |  |
| Mean PGIC Score | 1.7 ± 1.2 |  | 0.8 ± 1.3 |  | <0.001 |
| Proportion with ≥1 Minimally Improved PGIC | 83 (75/90) |  | 61 (53/87) |  | 0.001 |
| Proportion with ≥2 Much Improved PGIC | 63 (57/90) |  | 32 (28/87) |  | <0.001 |
| Proportion with ≥3 Very Much Improved PGIC | 31 (28/90) |  | 5.7 (5/87) |  | <0.001 |
| ***Composite Outcomes*** | | | |  |  |
| Proportion with ≥30% BPI5 and/or ≥30% ODI | 81 (73/90) |  | 60 (51/85) |  | 0.003 |
| Proportion with ≥30% BPI5 and ≥30% ODI | 62 (55/89) |  | 33 (28/85) |  | <0.001 |
| Proportion with ≥50% BPI5 and/or ≥50% ODI | 64 (58/90) |  | 38 (32/85) |  | <0.001 |
| Proportion with ≥30% BPI5 and/or ≥30% BPI9 | 83 (75/90) |  | 64 (56/87) |  | 0.006 |
| Proportion with ≥30% BPI5 and ≥30% BPI9 | 69 (61/88) |  | 37 (32/87) |  | <0.001 |
| Proportion with ≥50% BPI5 and/or ≥50% BPI9 | 67 (60/89) |  | 43 (37/87) |  | 0.001 |
| ***EQ-VAS*** | | | |  |  |
| Mean Point Change in EQ-VAS | 13 ± 22 |  | 5 ± 23 |  | 0.011 |
| Mean % Change in EQ-VAS | 31 ± 59 |  | 16 ± 46 |  | 0.024 |
| *Outcomes with continuous data (i.e., pt-change or % change) are shown ± standard deviation, with significance assessed between a two-way Wilcoxon rank-sum test; Outcomes with proportions (%, n/N): two-sided Fisher’s exact test. *p < 0.05; Abbreviations: BPI, Brief Pain Inventory; BPI3, Worst Pain Intensity; BPI5, Average Pain Intensity; BPI9, Pain Interference; EQ-VAS, EuroQol Visual Analog Scale; ODI, Oswestry Disability Index; PGIC, Patient Global Impression of Change; PNS, peripheral nerve stimulation;* | | | | | |

| **S U P P L E M E N T A R Y T A B L E S4.** Group #1 Compared to Group #2 Treatment Subgroups | | | |
| --- | --- | --- | --- |
|  | Group #2 Subgroup Success Rate  % (n/N) |  | p-value vs.  Group #1 Percutaneous 60-Day PNS^†^ |
|  |  |  |  |
| ***Interventional CLBP Treatments*** | | | |
| Lumbar Radiofrequency Ablation (RFA) | 26 (10/38) |  | 0.003 |
| Therapeutic Injections (ESI, Trigger Point, facet, SI, etc.) | 26 (9/34) |  | 0.005 |
| ***Other CLBP Treatments (e.g., Conservative Care)*** | | | |
| Other (e.g., used only physical therapy, chiropractic treatment, acupuncture, TENS, etc.) | 13 (1/8) |  | 0.026 |
| *Among participants receiving a treatment for CLBP, results are shown for the Primary Clinical Endpoint (i.e., proportion of participants experiencing substantial ≥50% pain relief at 3-months post-treatment) for the most commonly received treatment subgroups.* ^†^*Subgroup success rates were compared to the success rate for percutaneous 60-day PNS (56%, n=50/90) using a Fisher Exact Test. Abbreviations: ESI, epidural steroid injection; PNS, peripheral nerve stimulation; RFA, radiofrequency ablation; SI, sacroiliac joint;* | | | |

| **S U P P L E M E N T A R Y T A B L E S5.** Adverse Events Related to Percutaneous 60-Day PNS | | | |
| --- | --- | --- | --- |
| **Category of Adverse Event** | **Total Number of Adverse Events (n participants)** |  | **Rate of Occurrence^†^** |
| *Skin Irritation* | 41 (35) |  | 31.5% |
| *Pruritus (Itching)* | 22 (22) |  | 19.8% |
| *Pain* | 9 (9) |  | 8.1% |
| *Discomfort* | 6 (6) |  | 5.4% |
| *Infection, per lead exit site* | 6 (6) |  | 2.7% |
| *Granuloma, per lead exit site* | 3 (3) |  | 1.4% |
| *Discoloration* | 1 (1) |  | 0.9% |
| *Other: neurological/other (e.g., muscle spasms, stinging sensations)* | 4 (4) |  | 3.6% |
| *Other: cardiovascular (e.g., temporary vasovagal response)* | 1 (1) |  | 0.9% |
| *No serious, unanticipated study-related adverse events occurred for any participants in this study.* ^†^*Adverse events are shown for participants in Group #1 (n=111) included in the Safety Analysis Set. Rate of occurrence is reported per participant unless otherwise noted (i.e., per lead exit site). The 6 lead exit site infections were all superficial skin infections treated with a course of oral antibiotics; two subjects had leads removed and were exited from the study and the other four continued study participation with leads in place.* | | | |
